# Spinal Irritation

**Published:** 1833

**Authors:** Charles C. Hildreth

**Affiliations:** Zanesville, Ohio


### Art. III.—Spinal Irritation.

*An account of a case of Spinal Irritation, with Rheumatism and Hysteria, by* Charles C. Hildreth, M. D., *of Zanesville, Ohio.*

Mrs. K. H., aged 28, having recently returned from a malarious district of country, consulted me concerning an Intermittent, not long since contracted. I found her in the third paroxysm, with fever of a high grade, violent pain in the head and side, and acute sensibility to pressure on the false ribs of the left side.

Her pulse being full and strong, and her skin hot and dry, I directed venesection nearly to syncope, with much apparent advantage. Profuse diaphoretics followed the use of the lancet, with prompt alleviation of pain. Her bowels not having been moved for the last three days, I prescribed calomel 20 grains, and Dover's powder 10 grains, mixed, to be taken immediately, and followed in five hours by cathartic pills, till free purging took place. On instituting a more minute examination of her case, I ascertained that the type was quotidian; that she had recently recovered from a tertian of seven weeks duration, and that her general health was very much impaired. Her menses, she informed me, had been suppressed for the last two years. In the meantime she had been afflicted with the usual routine of hysterical symptoms; such as palpitation of the heart, globus hystericus, syncope, &c.

The succeeding paroxysm, attended with hysterical delirium, was so violent, that her attendants supposed her dying. For the purpose of inducing quietude, I gave her the usual antispasmodics, with the desired effect. I was then, for the first time, induced to examine her spine, which (as I anticipated) was found acutely sensible throughout nearly its whole extent. Pressure over the upper cervical vertebræ, very much increased the pain in the head, which being of a superficial character, and confined to the scalp externally, I at once perceived to be of spinal origin, consisting in a neuralgic affection of the sub-occipital and posterior branch of the first pair of cervical nerves. Pressure over the upper dorsal vertebræ very much aggravated the gastric distress, globus hystericus, &c. By pressing on the lower dorsal vertebræ, the pain and sensibility of the side were much increased. When I made pressure over the lumbar spine, the patient complained of acute pain in the knee, and wished me to desist. The joint being swollen and quite sensible to pressure, constituted a very fair case of rheumatism.

Having now perfectly satisfied my mind concerning the nature of the case, I at once proceeded to its radical treatment.

The upper dorsal being the most sensible part of the spine, I directed a blister to the six or eight upper vertebræ, and a turpentine embrocation to the remaining portions; and also exhibited ten grains of sulphate of quinine, during the febrile intermission, with the expectation of preventing the recurrence of the paroxysms, but was disappointed. The irritation of the cantharides, together with the previous depletion, (which was no doubt directly injurious,) gave rise to another hysteric paroxysm, attended with long continued syncope, and followed by a chill and slight fever; much more mild, however, than the preceding. The patient's stomach was also very irritable, frequently rejecting its contents. On the morning following, (Sept. 3d,) I found the blister discharging freely; stomach much less irritable, and hysterical symptoms much alleviated. Applied another blister over the lumbar spine, and directed 8 grains of sulphate of quinine, before the expected paroxysm. This had the effect of promoting its recurrence.

Sept. 4th. No pain has been felt in the side or knee; but acute pain in the head last evening, followed by profuse diaphoresis. The patient feels much debilitated. Directed 6 grains sulphate of quinine to be taken daily, and a blister to the cervical spine, for the relief of the headache, which was evidently neuralgic, as before explained; a more nourishing diet, and occasional stimulants.

From this time the patient improved rapidly. The blisters were kept discharging for several days, and were reapplied to the several tender portions of the spine, until all these anomalous, neuralgic, hysteric, and rheumatic affections disappeared. Her catamenia, if not finally suppressed, will probably be restored under the use of aloes, iron, and aromatics.

### Remarks.

This case I deem interesting, on account of its complicated character: presenting all the symptoms of spinal neuralgia, hysteria, rheumatism and intermittent fever, collectively. I need not say that all these symptoms depended upon disease of the spinal column, except, perhaps, the intermittent, which, primarily, at least, had a malarious origin. I apprehend, however, that much difficulty would have been found, in permanently curing the intermittent, without first removing the spinal irritation. Her previous attack of ague, she informed me, was not checked until a severe salivation had been induced; which continued to within a few days of her last attack. The rheumatism, which in this case was of a purely neuralgic character, constituted, perhaps, its most interesting feature. During her previous attack of the intermittent, she had, as she informed me, suffered rheumatism in the elbow and wrist; and that the pain was confined almost entirely to the paroxysm. The knee joint alone has been affected since she came under my charge. During the febrile exacerbation, she has complained much of the pain and tenderness of the joint, which was greatly increased by pressure upon the lumbar spine, over the origin of the affected nerves.

I think the profession much indebted to Dr. J. K. Mitchell, for his pathological and practical investigations, concerning the spinal origin and treatment of rheumatism.

To the prompt relief afforded by cups over the origin of the affected nerves, in acute rheumatism, I can testify from personal observation. And although a blister does not usually produce such obvious relief, as the abstraction of blood by cups, yet in the present case its action was indisputable. The tenderness of the spine, which I have never before observed in rheumatism, was in this case well marked; pressure over the origin of the affected nerves, very much increasing her sufferings; clearly proving, in this case, at least, the rheumatism to be of spinal origin. The acute pain and sensibility of the side, had, no doubt, a similar origin; being a neuralgic affection of the intercostal nerve. Pressure over the lower dorsal vertebræ, aggravated the pain; and the febrile paroxysm not only increased the pain, but rendered the integuments so sensible, that the patient could not bear the least pressure of my hand upon her side. The rheumatism, and this neuralgia of the side, were certainly strikingly analogous, and justify us in referring them to a common cause.

The hysterical symptoms were in this, as Dr. Tate supposes them to be in all cases, dependent on spinal disease.

Since the suppression of her menses, she had suffered very much from palpitation of the heart, syncope, delirium, and various kinds of gastric distress, such as flatulence, acidity, &c. From all these symptoms she obtained no permanent relief, until, by the repeated application of blisters, the spinal irritation was removed. It gives me pleasure to add, that the patient now enjoys much better health than she has had for some years past.

*Oct.* 5, 1833.

